# Association of Anemia with Parathyroid Hormone Levels and Other Factors in Patients with End-Stage Renal Disease Undergoing Hemodialysis: A Cross-Sectional, Real-World Data Study in Pakistan

**DOI:** 10.1155/2023/7418857

**Published:** 2023-02-13

**Authors:** Humera Bukhari, Aneeqa Ahmad, Amna Noorin, Aimal Khan, Mehwish Mushtaq, Aamir Naeem, Muhammad Rashid Iqbal, Faiza Naureen, Yasar Shah, Ahad Qayyum, Syed Munib, Amer Azhar, Farman Ullah, Faris Farooq Saeed Khan

**Affiliations:** ^1^Nephrology Ward, Jinnah Teaching Hospital, Khyber Pakhtunkhwa, Peshawar, Pakistan; ^2^District Headquarters DHQ Hospital, Faisalabad, Punjab, Pakistan; ^3^Peshawar Institute of Cardiology -M.T. I, Khyber Pakhtunkhwa, Peshawar, Pakistan; ^4^Northwest General Hospital and Research Center, Khyber Pakhtunkhwa, Peshawar, Pakistan; ^5^Department of Pharmacy, University of Peshawar, Khyber Pakhtunkhwa, Peshawar, Pakistan; ^6^Consultant Physician in Endocrinology, Madinat Zayed Hospital SEHA Abu Dhabi Health Services, Abu-Dhabi, UAE; ^7^Consultant Physician in Medicine, University Hospital Birmingham, UK; ^8^Department of Pharmacy, Abdul Wali Khan University Mardan, Khyber Pakhtunkhwa, Pakistan; ^9^Nephrology Ward, Bahria International Hospital, Lahore, Pakistan; ^10^Nephrology Ward, Institute of Kidney Disease Hayatabad Peshawar, Khyber Pakhtunkhwa, Peshawar, Pakistan; ^11^Nephrology Ward, Khyber Teaching Hospital M. T. I., Khyber Pakhtunkhwa, Peshawar, Pakistan; ^12^Department of Community Health Sciences, Aga Khan University, Karachi, Pakistan

## Abstract

End-stage renal disease (ESRD) patients are mostly managed with maintenance hemodialysis (MHD). ESRD patients on MHD also present with many complications, such as anemia, hyperparathyroidism, and hepatitis prevalence. This study depicts the real-world scenario of anemia among MHD and end-stage renal disease patients in the Pakistani population. A retrospective, multicentric, and real-world data analytical study was conducted at 4 dialysis centers in Pakistan. The study had a sample size of *n* = 342 patients on maintenance hemodialysis. The data were gathered from the medical records of patients. Data analysis was performed using STATA Version 16. Statistical significance was gauged at a 0.05 level of significance. According to our results, the mean age of the patients was 45 (±15) years. Most of the patients were male (*n* = 234, 68.4%), whereas 58.1% of the patients were maintained on twice-weekly hemodialysis. The most commonly reported comorbidities were hypertension and diabetes mellitus. The frequency of dialysis (*P* < 0.01) and comorbidities (*P* = 0.009) had a significant association with anemia in MHD patients. The majority of the patients had hyperparathyroidism (52%) with anemia. Upon performing binary logistic regression, multivariate analysis displayed a similar odds value for having anemia in patients with every additional month in the duration of hemodialysis (OR 1.01, *P* = 0.001), the odds of anemic patients having a positive antihepatitis-C antibody (OR 2.22, *P* = 0.013), and the odds of having anemia in patients in the age category below 45 years (OR 1.93, *P* = 0.013). In conclusion, the study results depict that every additional month in the duration of hemodialysis, age (<45 years), and positive anti-HCV antibody status, these variables were more likely to have anemia in our study MHD patients. While in our final multivariate model, no statistically significant association was observed between hyperparathyroidism and anemia.

## 1. Introduction

Anemia is one of the most common complications associated with maintenance hemodialysis (HD) patients. There are several reasons for developing anemia in end-stage renal disease (ESRD) patients. The reduction in erythropoietin (EPO) production is one of the main reasons for anemia in ESRD patients [1].

Anemia is considered to be one of the predictors of poor quality of life (pQOL), numerous morbidities, and mortality for maintenance HD (MHD) patients [2]. Some researchers reported that the mortality rate increases by 1.58 times with every 1 g/dL reduction in hemoglobin (Hb) levels [3]. Severe cardiac complications and alteration in cardiac output are the significant consequences of anemia that intensely deteriorate the quality of life of HD patients. Anemia can be defined as the hemoglobin (Hb) level becoming <12 g/dL in women and <13 g/dL in men [4]. Anemia is of multifactorial origin [5], and many researchers suggested the possible mechanisms for anemia in ESRD. The mechanisms suggested by researchers are as follows: iron deficiency, inflammation, and the raised parathyroid hormone (PTH) level also contribute to causing anemia [6].

**Figure 1:**
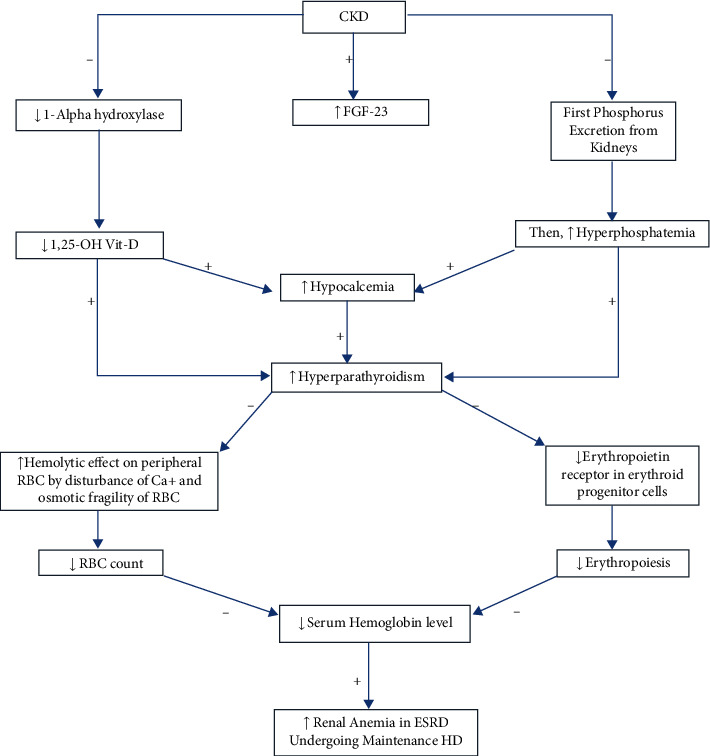
Schematic of the pathogenesis of renal anemia with hyperparathyroidism [8, 9]. CKD: chronic kidney disease; FGF-23: fibroblast growth factor 23; OH: hydroxy; vit-D: vitamin D; RBC: red blood cell; Ca+: calcium; ESRD: end-stage renal disease; HD: hemodialysis.

Renal anemia is a common complication with raised serum PTH levels in ESRD patients, and its pathogenesis (Figure 1) is complex and remains incompletely understood. Intact parathyroid hormone (iPTH) is mainly responsible for the regulation of calcium and phosphorus in blood, while calcium and phosphorus homeostasis is disturbed; that is, when calcium levels decrease in blood, phosphorus levels increase. This decreased calcium level due to deficiency of vitamin D (vit-D) or decreased mineral metabolism in ESRD patients eventually results in hypocalcemia and hyperphosphatemia. Hyperphosphatemia stimulates fibroblast growth factor 23 (FGF-23), a protein that is secreted by bones. First, FGF-23 increases the phosphorus excretion from the kidneys; later on, it further enhances hyperphosphatemia by retention of phosphorus from the kidneys [7]. Consequently, increased serum phosphorus binds with bioavailable calcium to produce calcium hydrogen phosphate (CHPO_4_), leading to hypocalcemia that stimulates PTH production and secretion. FGF-23 also decreases the conversion of the inactive form of vit-D to active form of vit-D by reduction of 1-alpha hydroxylase activity [7]. Decreased availability of the vitamin D active form leads to further hypocalcemia; as a result, it produces drastic increments in the iPTH levels and contributes to secondary hyperparathyroidism in ESRD patients [8].

Secondary hyperparathyroidism or renal hyperparathyroidism (rHPT) decreased the serum Hb level in ESRD patients by two mechanisms. During the first mechanism, the increased serum iPTH level leads to a significant decrease in erythropoietin production that causes an inhibitory effect on erythroid progenitors by downregulating erythropoietin receptors and increasing the insensitivity of the erythroid progenitor's cell to EPO. Consequently, reduced erythropoiesis and decreased serum Hb levels were observed in ESRD patients [9]. The increased serum iPTH level leads to a significant decrease in erythropoietin production. Many researchers have proved this hypothesis by demonstrating that parathyroidectomy reduces the EPO requirement in ESRD patients [4]. Several researchers reported that numerous EPO receptors on erythroid progenitor cells might be regulated by various factors such as calcitriol. The deficiency of calcitriol leads to downregulation of EPO receptors on the erythroid progenitor, and as a result, the erythroid progenitor becomes insensitive to the erythropoietin action. The second mechanism, increased iPTH, leads to a hemolytic effect due to calcium disturbance and enhances osmotic pressure and the fragility of red blood cells (RBCs). Both mechanisms lead to renal anemia in ESRD patients [9].

According to the “Kidney Disease Outcomes Quality Initiatives” (KDOQI) [10, 11] and the “Kidney Disease Improving Global Outcomes” (KDIGO) guidelines 2013 [12], chronic kidney disease (CKD) is defined as the incidence of kidney damage and deterioration of kidney function, irrespective of the cause, for ≥3 months. Secondary hyperparathyroidism is an insidious disease, and it is commonly associated with the early course of CKD and worsens with the decrease in the glomerular filtration rate (GFR) [13]. KIOQI recommendations for targeted serum iPTH levels according to CKD stages are as follows: in stage 3, the serum iPTH level should be within the range of 35 to 70 pg/mL, in stage 4, serum iPTH lies between 70 and 110 pg/mL, and in stage 5 or ESRD, serum iPTH should be between 150 and 300 pg/mL [11, 14]. In Pakistan, CKD affects 21.2% of the population [15], and a potential predictor >100 per million population of Pakistan annually undergoes ESRD [16]. Published studies have reported the association of anemia with hyperparathyroidism in MHD patients. Some studies have reported a negative correlation between Hb and iPTH [1, 17, 18], and another study has reported a weak negative correlation between Hb and iPTH levels [13]. However, their results regarding the correlation between PTH and serum Hb were not consistent. Till today, no research study has used a multivariate model to evaluate the association of Hb and iPTH levels and validate their associations in ESRD Pakistani patients. Also, all previously reported studies were conducted on a small pool of hemodialysis patients [1, 4, 13, 17, 18]. Most of the studies did not mention which recommended cutoff levels of hyperparathyroidism (iPTH > 300 pg/mL) were used for the determination of anemia associated with raised iPTH levels [1, 18]. The existing literature is insufficient for healthcare professionals in Pakistan to devise local recommendations for the clinical prognosis and treatment modalities in patients with raised iPTH undergoing maintenance hemodialysis.

Besides this, there was a need to conduct a study on a large group of maintenance hemodialysis patients to validate the association between hyperparathyroidism and anemia. Therefore, the rationale of our study is to evaluate the prevalence of anemia in ESRD-undergoing MHD patients and the correlation between serum Hb levels with raised intact PTH (iPTH > 300 pg/mL) levels and other factors. The attained information would be beneficial to healthcare professionals and health education programs for better management of renal anemia with secondary hyperparathyroidism in ESRD patients undergoing MHD.

## 2. Materials and Methods

### 2.1. Study Design and Clinical Settings

A retrospective, multicentric, and real-world data analytical study was conducted from January 2021 to May 2022 at the dialysis centers of Lahore, Faisalabad, and Peshawar in Pakistan. The data were collected from patient files of institutes that include District Headquarter (DHQ) Hospital, Faisalabad; Bahria Town International Hospital (BTIH), Lahore; Khyber Teaching Hospital (KTH), Peshawar; and the Institute of Kidney Diseases (IKD), Peshawar. DHQ, Faisalabad, KTH, Peshawar, and IKD, Peshawar, are public sector tertiary care teaching hospitals, while BTIH, Lahore, is a private sector tertiary care hospital.

### 2.2. Sample Size and Selection Criteria

The sample size (*n* = 342) was calculated by using the OpenEpi version 3, sample size calculator, keeping power at 80%, level of sig-nificance at 5%, and confidence interval at 95% [4, 18]. Male and female patients ≥18 years of age who had been on HD maintenance for at least 3 months were included in the study. Nonnative origin HD patients, pregnant HD patients, patients who had three months of bleeding history before initiation of the study, and patients with transplant workups were excluded from the study.

### 2.3. Ethical Approval

The study was conducted from January 2021 to May 2022 in multiple dialysis centers in Pakistan. The study was approved by the Ethical Review Committee (ERC) of Faisalabad Medical University, Faisalabad, with approval reference number (F.48-ERC/2021-22/PHRC/FMU/260) presented as (SF-1: appendix I).

### 2.4. Data Collection and Screening

**Figure 2:**
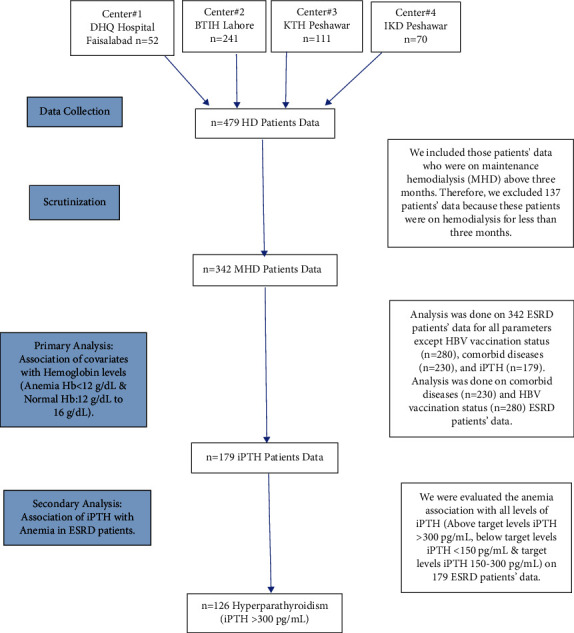
Flow diagram of data collection and scrutinization of end-stage renal disease ESRD patients' data. DHQ: District Headquarter; BTIH: Bahria Town International Hospital; KTH: Khyber Teaching Hospital; IKD: Institute of Kidney Diseases; HD: hemodialysis; MHD: maintenance hemodialysis; iPTH: intact parathyroid hormone; ESRD: end-stage renal disease; Hb: hemoglobin; HBV: hepatitis B.

The data were collected from the medical records of hemodialysis patients in the abovementioned sites, and screening was conducted based on the inclusion/exclusion criteria of the study. Upon successful screening, the data were filled in the predesigned proforma (SF-2: appendix II), which was later entered into a computer. The study included the natives of urban and rural areas of Pakistan who were on MHD. The data gathered in the study include demographics, date of 1^st^ dialysis or month of dialysis, number of HD per week, vascular access, vaccination for hepatitis B (HBV), cause of renal failure, any other comorbid disease, antihepatitis B surface agent (HBsAg), antihepatitis C (HCV) antibodies, systolic and diastolic blood pressure, and lab profiles (if available). For our study of ESRD patients, a cutoff value for serum iPTH categorized as <150 pg/mL represented below target serum iPTH levels and 150 to 300 pg/mL represented a targeted serum iPTH level, and for above target serum iPTH levels or hyperparathyroidism, we took a cutoff value (hyper-iPTH; >300 pg/mL); the specific ranges were referred from KIDGO guidelines [11, 13, 14]. Similarly, a cutoff value for hemoglobin in terms of anemia was referred from the “KDIGO Clinical Practice Guideline for Anemia in CKD” [10, 12, 19], below 12 g/dL represented anemia and 12 g/dL to 16 g/dL represented without anemia. The overall workflow of data collection and analysis is shown in Figure 2.

### 2.5. Statistical Analysis

Data analysis was accomplished using STATA version 16. For continuous variables, summary statistics (including the number of patient profile data (*n*), mean, standard deviation, median, minimum, and maximum values, as well as frequencies and percentages for categorical variables) were reported. Fisher's exact chi-square test was performed for categorical variables to gauge the statistical significance of the association between different factors and anemia. Cramer's test was performed to identify the correlation of all variables with serum Hb levels and serum iPTH levels. Statistical significance was gauged at *α* = 0.05 level of significance. Binary logistic regression was performed (*n* = 342), at both univariate and multivariate levels using the level of significance = 0.05. The final model was synthesized using statistically significant covariates from the univariate analysis with a forward approach; the odds ratio and *P* values were reported for the final model as well.

## 3. Results

**Figure 3:**
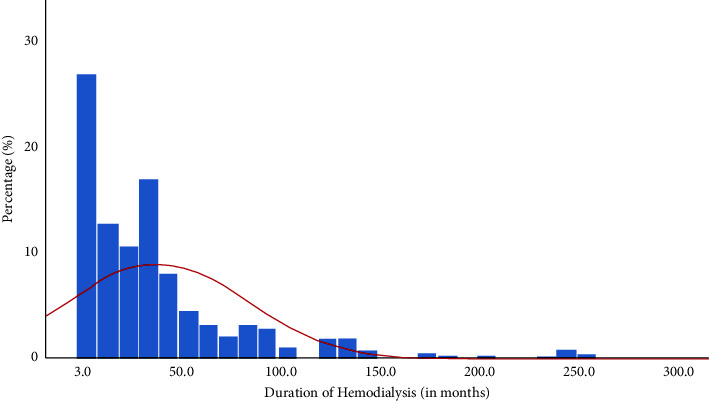
Graphical representation of the frequency of months on dialysis among our HD study patients.

Table 1 demonstrates the results; a total of 342 hemodialysis patients were recruited in the study, of which *n* = 234 (68.4%) were male and *n* = 108 (31.6%) were female. The mean age of the study patients was 45 ± 15 years. Our results showed that, out of *n* = 342 study patients, the frequency of dialysis was found to be more distributed as twice-weekly among *n* = 198 (58.1%) patients as compared to once or thrice weekly. A graphical representation of the month of duration of dialysis among our study HD patients is presented in Figure 3. Of the total serum Hb levels of 342 patients recorded, the analysis depicted that prevalence of anemia was higher in patients (*n* = 267, 78%) than that in patients (*n* = 75; 22%) having normal serum Hb levels. Among the patients, 42% were found to be anti-HCV antibodies positive. Our results showed a hepatitis B vaccination rate (*n* = 185, 66%) in the included study patients, while *n* = 12 patients (3.5%) were found to have HBV surface antigens (HBsAg). According to our results, the most common comorbidities observed among 230 HD patients were hypertension (*n* = 185, 80.4%) and diabetes mellitus (*n* = 23, 10%). Serum iPTH levels were reported for only 179 patients; however, out of those 179 patients, *n* = 126 patients (70%) were found to have hyperparathyroidism in our study MHD patients.

Table 2 presents the study correlation results for covariates of hemodialysis patient profiles with hemoglobin levels. Both age categories have a positive correlation (0.147) with serum Hb levels among studied MHD patients. In these age categories, they were also found to have a significant (*P* = 0.009) association with anemia. Both male and female hemodialysis patients had a nonsignificant (*P* = 0.237) association with anemia. Furthermore, the association of serum iPTH levels with serum Hb levels was also evaluated. Based on the statistical evaluation, raised serum iPTH levels were found to have a significant (*P* = 0.005) association with anemia as the majority of patients (*n* = 93, 52%) with hyperparathyroidism had anemia. Likewise, hepatitis C had a significant (*P* < 0.01) association with anemia as the majority of patients (*n* = 94, 27.5%) with anti-HCV antibodies positive had anemia. However, hepatitis B had a nonsignificant (*P* = 0.076) association with anemia, and only *n* = 12 (3.5%) patients were anemic with positive HBV surface antigens. As per the results, both comorbid diseases and the frequency of dialysis per week have a positive correlation with anemia among hemodialysis patients. The frequency of comorbid diseases with anemia in HD patients observed in this study was that *n* = 137 (59%) patients were anemic with hypertension and *n* = 20 (8.7%) patients were anemic with diabetes mellitus. In addition, the statistical test result shows (Table 2) a significant association between anemia with comorbid disease (*P* = 0.009) and the frequency of dialysis per week (*P* < 0.01).

Table 3 depicts the Fisher's exact statistic and chi-square test results that show a statistically insignificant (*P* = 0.106) association between anemia with serum iPTH levels based on stratification of age categories 18–44 and above 45 years in HD patients.

In our univariate model, serum Hb levels were kept as the dependent variable and the level of significance was at 0.250; the covariates such as frequency of dialysis per week, anti-HCV antibodies, age category, and months on hemodialysis were all statistically significant when univariate binary logistic regression was performed. All other variables except gender were excluded from the final model for the association of covariates with serum Hb levels, as reflected in Table 4.

In our final model for binary logistic regression, keeping hemoglobin levels as our dependent variable, we found that the odds of having anemia were similar to the odds of having normal hemoglobin levels, for every additional month on hemodialysis (*P* = 0.011).

Among our patients, the odds of having anemia were 2.2 times higher than the odds of having normal hemoglobin levels for those having a positive anti-HCV antibody status (*P* = 0.013), whereas when stratified based on the age group, the odds of having anemia were 1.93 times higher than the odds of having normal serum Hb levels, for patients aged between 18 and 44 years (*P* = 0.031), as reflected in Table 5. No significant interaction and confounding were found in the multivariate model. For the goodness of fit of a model, the Hosmer–Lemeshow test was performed, yielding a chi-square value of 8.95 (*P* = 0.346), signifying that the final selected model was not a poor fit.

## 4. Discussion

Anemia is one of the most common complications associated with HD maintenance patients. The present study has gauged anemia in ESRD patients on HD maintenance and determined the correlation of anemia with all covariates like demographics, hyperparathyroidism, and comorbid diseases in 342 maintenance HD patients in Pakistani numerous hemodialysis centers.

As per our results, the mean age of Pakistani ESRD patients on maintenance HD is 45 (±15) years. The two local Pakistani studies by Sabir et al. [20] and Qureshi et al. [21] also reported similar results. They reported the average age of HD patients as 48.56 (±19.15) and 48.71 (±11.92) years, respectively. Another study by Gamal et al. [1] reported the mean age of ESRD patients to be 52.23 (±15.37) years. The association of age categories with anemia is also comparable to the published data by Azeem et al. [4], which reported the frequency of anemia as 38.1% and 24.5% among 45–70 and 20–44 years in HD patients, respectively. However, according to the multivariate model in our study, the age category of 18–44 years has a significant (*P* = 0.031) association with anemia. In our results, young and adult-aged HD patients have more anemia than older HD patients in our study. It is an alarming situation for the Pakistani people that most young and adult-aged people who were to undergo chronic kidney disease have also faced severe anemia during MHD, according to our findings.

As per our study results, 52% of male patients and 25% of female patients were anemic, indicating a positive but nonsignificant correlation with anemia. Similar results are observed in the literature, as Lizardi Gómez et al. [6] report that the male gender is an independent risk factor for anemia.

The present study clearly emphasizes the occurrence of secondary hyperparathyroidism as one of the significant factors for persistent anemia among maintenance HD patients. It is evident from the literature that, among ESRD patients, the overproduction of PTH leads to changes in mineral and bone composition, eventually resulting in anemia [17]. Parathyroidectomy in uremic patients has improved the hematocrit levels due to an increase in serum concentration of immunoreactive erythropoietin which is an indirect piece of evidence of the correlation of PTH with anemia in ESRD patients [22]. As per our study, the association of raised serum iPTH levels with serum Hb levels was significant; however, the univariate regression analysis showed a non-significant association between hyperparathyroidism (serum iPTH level > 300 pg/mL) and anemia. Our results were comparable with a study by Adhikary et al. [13] that depicted a nonsignificant association between serum PTH and Hb levels in HD patients.

In the present study, the overall prevalence of anti-HCV antibodies positive was 42% among HD patients, which is comparable to previously published data by Akhtar et al. in 2021 [23] that found the prevalence of HCV infection in the Pakistani population as 32.33% among HD patients. Another researcher Lodhi et al. [24] also reported that the prevalence of HCV was 43.2% among HD patients in Quetta, Pakistan. As per our results, positive anti-HCV antibodies have a positive correlation with anemia; likewise, HCV is also a significant risk factor for anemia in our ESRD study population. The possible mechanism for a positive association of anemia with HCV in maintenance HD may be due to the negative impact of antiviral therapy, aplastic anemia caused by bone marrow suppression, and liver failure leading to kidney disease, which further causes anemia in HCV-positive patients [25, 26]. Our results are in agreement with Elnaggar et al.'s [27] study, in which they demonstrated that slightly higher mean Hb levels were found in HCV-positive (Hb = 8.02 ± 1.08 g/dL) HD patients than in HCV-negative (Hb = 7.64 ± 2.15 g/dL) HD patients, but anemia persisted in their results.

Furthermore, our results showed an overall prevalence of Positive-HBV surface antigen among study HD patients, which was found to be comparable with Jeele et al. [28] study.According to our study results, hepatitis B has a non-significant association with anemia, which is also comparable with the data published by Lodhi et al. [24] and Elnaggar et al. [27].

Both comorbid diseases, hypertension and diabetes mellitus, have a significant association and positive correlation with anemia among hemodialysis patients, which is comparable with the data published by Junejo et al. [18] and Alshogran et al. [29] study results.

Most of our study patients were on twice-per-week dialysis, depicting a significant association with anemia, which is also comparable with the published data by Sabir et al. [20], in which twice-per-week dialysis was observed in 75% of study patients. The possible cause of anemia with the increased frequency of dialysis per week may be blood loss, which usually gets retained in the dialyzer machine [30]. Furthermore, our study results have represented the duration of hemodialysis and evaluated the association of anemia with every additional month in the duration of hemodialysis in ESRD patients. However, previously reported studies did not identify the association of anemia with every additional month of hemodialysis in ESRD patients in Pakistan.

Anemia is a significant contributing factor for the mortality rate among ESRD patients. Therefore, we identified the correctable or uncorrectable factors of anemia in ESRD patients in Pakistan and also validated our results from multivariate analysis, whereas most researchers identified only correctable factors and did not validate their results by a multivariate analysis model [4, 18].

## 5. Study Limitations

Being a real-world data gathering study, this study has some limitations: (i) it is a small sample size study, (ii) there are a smaller number of variables incorporated in the study because of a lack of data availability, (iii) it is not quite a multicentric study (it is suggested that more multicentric studies should be performed on the Pakistani population with large data to have generalizable results), and (iv) finally, financial constraints have also had an impact on availability of expensive lab test (such as ferritin, albumin, interleukin 1, and interleukin 6) data in whole.

## 6. Conclusions

In our results, we found a higher prevalence of anemia among ESRD patients with maintenance HD. Based on our final multivariate model, we conclude that there was no statistically significant association found between anemia and hyperparathyroidism. Moreover, the study results depict that every additional month in the duration of hemodialysis, having age (<45 years), and positive anti-HCV antibody status, these variables were more likely to have anemia in our study MHD patients.

## 7. Recommendations

(i) Our results highlighted the clinical relevance of anemia with a positive anti-HCV antibody status and every additional month in the duration of hemodialysis in ESRD patients. So anemia in ESRD patients could be improved by correcting hepatitis-C status and dialyzer machine fluid composition. (ii) Because this study is real world and retrospective in nature, further prospective study and genomic study are required for further investigations of unexplained reasons for less than forty-five years of age ESRD patients being more anemic. (iii) Further studies are necessary for assessing the correlation of medications (such as erythropoietin stimulating agents, calcium, and vit-D supplementation) with anemia status in ESRD patients.

## Figures and Tables

**Table 1:** Demographic characteristics of patients (*n* = 342)^∗^.

Demographics characteristics		Mean (±SD), *n* (%)
Age (in years)		45 (±15)
Gender^∗^	Male	234 (68.4%)
	Female	108 (31.6%)

Dialysis per week^∗^	Once	16 (4.4%)
	Twice	198 (58.1%)
	Thrice	128 (37.5%)

Hemoglobin levels∗ (g/dL)	Anemia (Hb < 12 g/dL)	267 (78%)
	Normal (Hb12 g/dL to 16 g/dL)	75 (22%)

Anti-HCV antibodies^∗^	Negative	198 (58%)
	Positive	144 (42%)

HB surface antigen^∗^	Negative	330 (96.5%)
	Positive	12 (3.5%)

Comorbid diseases (*n* = 230)	None	22 (9.6%)
	Diabetes mellitus	23 (10%)
	Hypertension	185 (80.4%)

Intact parathyroid hormone (pg/mL) (179)	Below target levels (iPTH < 150 pg/mL)	11(6.1%)
	Target levels (iPTH 150–300 pg/mL)	43 (23.9%)
	Above target levels or hyperparathyroidism (iPTH > 300 pg/mL)	126 (70%)

HBV vaccination status (*n* = 280)	Yes	185 (66%)
	No	95 (34%)

**Table 2:** Association of covariates with hemoglobin levels.

Variables		Hemoglobin levels		*P* value^∗^	Correlation^∗∗^
		Anemia (Hb < 12 g/dL)	Normal (Hb:12 g/dL to 16 g/dL)		
Age categories	18 – 44 years	114 (33.3%)	48 (14%)	0.009^∗∗∗^	0.147
	45 years and above	153 (44.7%)	27 (8%)		

Gender	Male	178 (52%)	56 (16.4%)	0.237	0.062
	Female	89 (25%)	19 (5.6%)		

Intact parathyroid hormone (pg/mL)	Below target levels (iPTH <150 pg/mL)	9 (5%)	2 (1.1%)	0.005^∗∗∗^	0.221
	Target level (iPTH 150–300 pg/mL)	41 (23%)	2 (1.1%)		
	Above target levels or hyper (iPTH > 300 pg/mL)	93 (52%)	32 (17.8%)		

HBV surface antigen	Positive	12 (3.5%)	0 (0%)	0.076	0.101
	Negative	255 (74.5%)	75 (22%)		

Anti-HCV	Positive	94 (27.5%)	50 (14.6%)	<0.01^∗∗∗^	0.272
	Negative	173 (50.6%)	25 (7.3%)		

Comorbid	None	22 (10%)	0 (0%)	0.009^∗∗∗^	0.193
	Hypertension	137 (59%)	48 (21%)		
	Diabetes mellitus	20 (8.7%)	3 (1.3%)		

Number of dialysis (per week)	Once	14 (4.1%)	1 (0.3%)	<0.01^∗∗∗^	0.371
	Twice	176 (51.6%)	22 (6.5%)		
	Thrice	76 (22.3%)	52 (15.2%)		

^∗^Fisher's exact chi-square test was performed. ^∗∗^Cramer's V correlation. ^∗∗∗^ statistically significant.

**Table 3:** Association of hemoglobin levels with PTH based on age categories.

Hemoglobin levels		Intact parathyroid hormone (pg/mL)				*P* value^∗^	Correlation^∗∗^
		Below target levels (iPTH < 150 pg/mL)		Target level (iPTH 150–300 pg/mL)	Above target levels or hyper (iPTH > 300 pg/mL)		
Anemia (Hb < 12 g/dL)	Age categories	18–44 years	5 (3.5%)	12 (8.4%)	44 (30.8%)	0.106	0.176
		45 and above	4 (2.8%)	29 (20.3%)	49 (34.2%)		

Normal	Age categories	18–44 years	0 (0%)	1 (2.7%)	20 (55.5%)	0.310	0.292
		45 and above	2 (5.2%)	1 (2.7%)	12 (33.3%)		

^∗^Fisher's exact statistic chi-square test was performed. ^∗∗^Cramer's V correlation coefficient.

**Table 4:** Univariate model of covariates with hemoglobin levels.

Variable		Odds ratio	95% CI	*P* value
Gender	Male	1.47	0.82–2.63	0.189^∗∗^
	Female	—	—	—

Dialysis per week	Once	0.10	0.01–0.81	0.032^∗∗^
	Twice	0.18	0.22–13.9	<0.001^∗∗^
	Thrice	—	—	—

Anti-HCV antibodies	Negative	—	—	—
	Positive	3.68	2.14–6.32	<0.001^∗∗^

HBV surface antigen	Negative	—	—	—
	Positive	1.00	X	x

Comorbid diseases	None	—	—	—
	Diabetes mellitus	1.00	—	—
	Hypertension	2.35	0.67–8.27	0.146

Intact parathyroid hormone (pg/mL)	Below target levels (iPTH < 150 pg/mL)	—	—	—
	Target levels (iPTH 150–300 pg/mL)	0.21	0.03–1.77	0.155
	Above target levels or hyperparathyroidism (iPTH > 300 pg/mL)	1.54	0.31–7.54	0.589

HBV vaccination status	Yes	0.92	0.52–1.63	0.785
	No	—	—	—

Age categories	18–44 years	—	—	—
	45 years and above	0.41	0.24–0.71	<0.001^∗∗^

Months on hemodialysis		1.01	1.00–1.02	<0.001^∗∗^

^∗^Categories are reference categories. ^∗∗^ = statistically significant, level of significance = 0.250.

**Table 5:** Multivariate model for association of covariates with hemoglobin levels.

Variable		Odds ratio	95% CI	*P* value
Months on HD		1.00	1.00–1.01	0.011^∗∗^

Dialysis number	Twice	1.08	0.13–8.93	0.940
	Thrice	3.42	0.40–28.75	0.256

Anti-HCV antibodies	Positive	2.22	1.18–4.18	0.013^∗∗^

Age categories	18–44 years	1.93	1.06–3.51	0.031^∗∗^

Gender	Male	1.47	0.82–2.63	0.189

^∗∗^ = statistically significant, level of significance = 0.05.

## Data Availability

All relevant data are within the paper and their supporting information files. The statistical analysis excel sheet data used to support the findings of this study are available from the corresponding author upon request.

## Supplementary Materials

Supplementary Materials SF-1: appendix-I: it is an Ethical Review Committee (ERC) of Faisalabad Medical University approval document.SF-1: appendix-Ia: it is a Khyber Teaching Hospital Ethical Review Committee (ERC) approval document. SF-2: appendix-II: it is a predesigned proforma in which data were filled from patients' record file.
