# Supplementary material for: Association of Anemia with Parathyroid Hormone Levels and Other Factors in Patients with End-Stage Renal Disease Undergoing Hemodialysis: A Cross-Sectional, Real-World Data Study in Pakistan
Source: Int J Clin Pract. 2023 Feb 13;2023:7418857. doi: 10.1155/2023/7418857 (PMC9940945; doi:10.1155/2023/7418857)
Supplement: Supplementary Materials — SF-1: appendix-I: it is an Ethical Review Committee (ERC) of Faisalabad Medical University approval document.SF-1: appendix-Ia: it is a Khyber Teaching Hospital Ethical Review Committee (ERC) approval document. SF-2: appendix-II: it is a predesigned proforma in which data were filled from patients' record file. [file 7418857.f1.zip › SF-1 Appendix Ia ERC Document (1).pdf]

Khyber Teaching Hospital Peshawar

University Road, Peshawar Phone:091-9224400
Email: Info@kth.gov.pk, Website: http// www.kth.gov.com

Hospital Ethical Committee

Dated 6th April 2022

CERTIFICATE OF APPROVAL

Member of the Institution Ethical Review Committee have evaluated the post graduate Research synopsis / survey

" **Evaluate the prevalence of Anemia with raised Parathyroid hormones(PTH) level in Hemodialysis patients: A cross-sectional multicentre study in Pakistan**" including District Head quarter Hospital Faisalabad, Bahria Town International Hospital Lahore, Institute of Kidney Disease Peshawar, Study is approved by ethical Review committee for a period of 6 months.

During the whole work, all personal information of patients/ subjects should be kept confidential.

Asso. Prof Dr Ahmad Zeb Khan

Member Hospital Ethical committee

Khyber Teaching Hospital

Peshawar

Scanned with CamScanner
