# Supplementary material for: Association of Anemia with Parathyroid Hormone Levels and Other Factors in Patients with End-Stage Renal Disease Undergoing Hemodialysis: A Cross-Sectional, Real-World Data Study in Pakistan
Source: Int J Clin Pract. 2023 Feb 13;2023:7418857. doi: 10.1155/2023/7418857 (PMC9940945; doi:10.1155/2023/7418857)
Supplement: Supplementary Materials — SF-1: appendix-I: it is an Ethical Review Committee (ERC) of Faisalabad Medical University approval document.SF-1: appendix-Ia: it is a Khyber Teaching Hospital Ethical Review Committee (ERC) approval document. SF-2: appendix-II: it is a predesigned proforma in which data were filled from patients' record file. [file 7418857.f1.zip › SF-2 Appendix II Data Collection Proforma.pdf]

Association of anemia with parathyroid hormone levels and other factors in patients with end-stage renal disease undergoing hemodialysis: A cross-sectional, real-world data study in Pakistan.

Name of Center ______________________________ ______________

Phone Number ________________________ Sex ☐M ☐F DOB / Age

CNIC ______________

Address (only city) ________________________; Patient Type: New, Old

Date of 1st dialysis or months on dialysis ________________________

No of HD / Week: Once, Twice, Thrice

Patient Status On ________ HD, Transplant Workup, Expired, Transfer to other center

Vascular Access: Central Line, AV Fistula, AV Graft, Perm Cath

____

Vaccination of HBV Yes / NO HD Sponsored by: Self, Department, Govt. ONGO / Trust, Other

____

Cause of renal failure: DM, H TN, Ch. GN, Stone, Polycystic, Other

____

Any other disease

____ DM ☐HTN ☐IHD ☐TB ☐ROD ☐Other ________________

Anti HCV: +ve, -ve if positive then how many months on HD HCV by PCR ________ ☐+ve ☐-ve

HBsAg: +ve, -ve if positive then how many months on HD HBsAg by PCR ________ ☐+ve ☐-ve

Systolic Blood Pressure ________________ Diastolic Blood Pressure ________________

Hb ______ TLC ______ Plt ______ BUN ______ Cr ______ T. Bil ALT AST

ALP PTH BSL(F) BSL(R) Ca Phos ALB

________ ________ ________ ________ ________ ________ ________
