# Supplementary figures and images for: Association of Anemia with Parathyroid Hormone Levels and Other Factors in Patients with End-Stage Renal Disease Undergoing Hemodialysis: A Cross-Sectional, Real-World Data Study in Pakistan
Source: Int J Clin Pract. 2023 Feb 13;2023:7418857. doi: 10.1155/2023/7418857 (PMC9940945; doi:10.1155/2023/7418857)

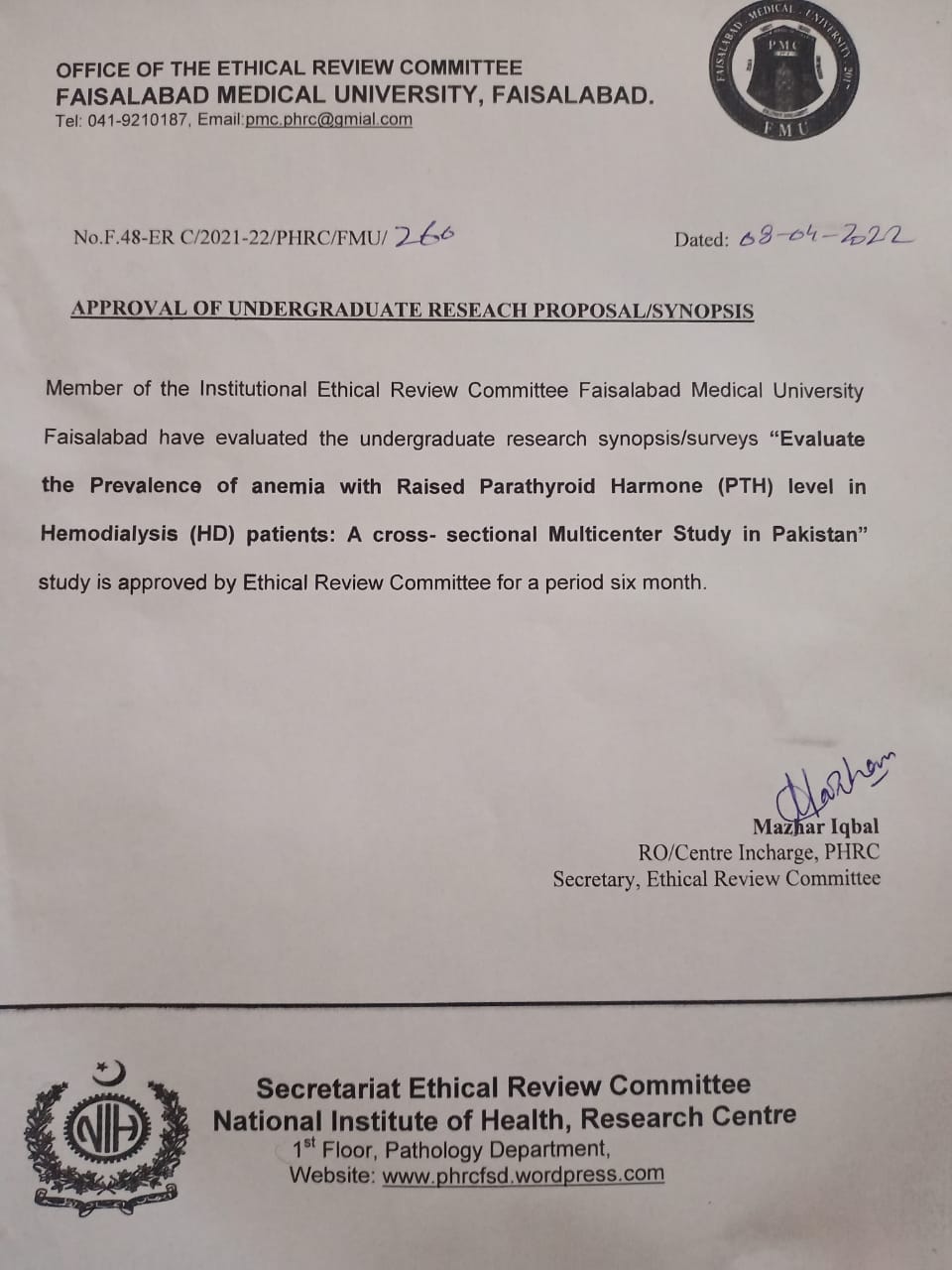

Supplement: Supplementary Materials — SF-1: appendix-I: it is an Ethical Review Committee (ERC) of Faisalabad Medical University approval document.SF-1: appendix-Ia: it is a Khyber Teaching Hospital Ethical Review Committee (ERC) approval document. SF-2: appendix-II: it is a predesigned proforma in which data were filled from patients' record file. [file 7418857.f1.zip › SF-1 Appendix I ERC Document.jpg]
